# Right ventricular thrombus after penetrating cardiac injury

**DOI:** 10.1186/1749-8090-8-S1-P33

**Published:** 2013-09-11

**Authors:** D Nenadić, C Bulat, D Kocen, M Lojpur, Z Marović, M Milić, D Oršulić, J Vojković, V Ćorić, D Letica

**Affiliations:** 1 Department of Cardiac Surgery, School of Medicine, University Hospital Centre Split, Croatia; 2 Department of Anesthesiology, School of Medicine, University Hospital Centre Split, Split, Croatia

## Background

To report a case of thrombus formation ten days after penetrating cardiac injury to the right ventricle.

## Methods

A 37 year old man had been stabbed with a knife to the epigastric area. Twenty minutes after the incident he was admitted to the University Hospital Centre Split. After aggressive resuscitation the patient was transferred to emergency operating theatre without any prior diagnostic procedures. Abdominal cavity was explored by laparotomy, and no evident damage to the abdominal organs was found. Because of hemodynamic instability and high suspicion of cardiac tamponade a left thoracotomy was performed and the stab wound measuring 40mm in length to the right ventricle was located 20mm away from middle third of the left anterior descending artery. Because of space restrictions it was decided to do a middle sternotomy. The entry wound was explored and sutured with two pledgeted 4.0 polypropylene sutures, without the support of the heart-lung machine.

## Results

Patient spent two days in intensive care unit. After ten days of hospital stay the patient was discharged with normal echocardiography findings. After three weeks a control transthoracic echocardiography was performed and a right ventricle thrombotic mass measuring 27mm x 19mm x 13mm was detected. Immediately after that find the patient was treated with enoxaparine (low molecular weight heparin) for ten days.

At control magnetic resonance imaging (MRI) no thrombotic mass was found in the right ventricle.

## Conclusion

Out of this case it is evident that patients with penetrating cardiac injuries should to be intensively monitored by echocardiography during the first few weeks after injury, due to high risk of thrombotic mass formation at the repaired wall of the myocardium.

